# Breakdown Performance and Partial Discharge Development in Transformer Oil-Based Metal Carbide Nanofluids

**DOI:** 10.3390/nano12020269

**Published:** 2022-01-14

**Authors:** Konstantinos N. Koutras, Sokratis N. Tegopoulos, Vasilios P. Charalampakos, Apostolos Kyritsis, Ioannis F. Gonos, Eleftheria C. Pyrgioti

**Affiliations:** 1 High Voltage Laboratory, Department of Electrical & Computer Engineering, University of Patras, GR 26500 Patras, Greece; e.pyrgioti@ece.upatras.gr; 2 School of Applied Mathematics & Physical Sciences, National Technical University of Athens, GR 15780 Athens, Greece; stegopoulos@mail.ntua.gr (S.N.T.); akyrits@central.ntua.gr (A.K.); 3 Department of Electrical & Computer Engineering, University of the Peloponnese, GR 26334 Patras, Greece; charalambakos@uop.gr; 4 School of Electrical & Computer Engineering, National Technical University of Athens, GR 15780 Athens, Greece; igonos@cs.ntua.gr

**Keywords:** insulating nanofluid, SiC nanoparticles, AC breakdown voltage, partial discharge, relative permittivity, electrical conductivity

## Abstract

In this work, the influence of semi-conductive SiC nanoparticles on the AC breakdown voltage and partial discharge development in natural ester oil FR3 is examined. Primarily, the dielectric constant and the electrical conductivity of the nanoparticles are measured following the broadband dielectric spectroscopy technique. The nanoparticles are added into the matrix following the ultrasonication process in three weight percentage ratios in order for their effect to be evaluated as a function of their concentration inside the base oil. The processing of the results reveals that the nanofluid containing SiC nanoparticles at 0.004% *w*/*w* demonstrates the highest AC dielectric strength improvement and shows the greatest resistance to the appearance of partial discharge activity. The mechanisms behind the aforementioned results are discussed in detail and confirmed by the broadband dielectric spectroscopy technique, which reveals that this particular nanofluid sample is characterized by lower dielectric constant and electrical conductivity than the one with double the weight percentage ratio.

## 1. Introduction

One of the major issues that need to be solved in the context of the smart grid of the future is to ensure the transmission and distribution of electricity taking into account the stringent regulations along with environmental protection [1]. At these levels, the continuous operation of the power transformer is an urgent need, while its cost is calculated to be about 60% of the total substation cost [2,3]. Statistics have shown that the major aspects that lead to the limitation of the power transformer lifespan, and ultimately its failure, are based on insulation, which is mainly made of a combination of dielectric liquid and insulating paper impregnated in it [4,5,6,7]. In contrast to the mineral oils used conventionally, natural ester oils (NEO) are characterized by unique and preferable properties from the industry side, such as rapid biodegradability, high moisture tolerance, less flammability and increased lifetime in comparison to mineral oils [8,9,10,11,12]. Apart from that, their higher relative permittivity is also very important, as it can match that of solid insulation in order to limit electric stress in high loading situations [13].

On the other hand, there are serious drawbacks that still limit the widespread use of NEO as dielectric fluid in high-voltage (HV) equipment. Vegetable oils (VO) are characterized by high viscosity, which could lead to greater hotspot temperatures inside the HV equipment [14,15] and increased dielectric dissipation factor, which means augmented dielectric losses [16]. Therefore, for the sake of disregarding these suspensive factors and, in general, enhancing the dielectric and physicochemical characteristics of the NEOs, the focus of research is on the integration of a proper amount of nanoparticles (NPs) inside the NEO volume, with the resulting mixture called nanofluid (NF). NFs have also been used in other sectors [17,18,19], but in liquid insulation they are investigated in order to enhance the dielectric and physicochemical properties of the traditional oils [20,21,22,23,24,25,26,27,28,29,30,31,32]. NPs are classified based on their shape, size, type and electrical properties, namely conductive, semi-conductive and insulative.

With regard to their type, the influence of metal oxide NPs, i.e., TiO_2_, Al_2_O_3_, ZnO, Fe_2_O_3_ and Fe_3_O_4_, on the dielectric and thermal performance of biodegradable oils has been thoroughly investigated [8,11,12,21,22,23,24,25,26]. Olmo et al. [21] reported that the addition of TiO_2_ NPs (10–20 nm size) resulted in AC breakdown voltage (AC BDV) enhancement by 33.2% at a volume percentage ratio of 0.5 kg/m^3^, while in different concentrations the enhancement ranged from 7.6 to 30.4%. Khaled and Beroual [22] found that the addition of conductive Fe_3_O_4_ NPs with a diameter of 50 nm in synthetic ester oil (SEO) can lead to an increment in terms of AC BDV up to 48%. The same authors [23] reported that the integration of the same kinds of NPs in NEO can lead to a 7.51% maximum enhancement of negative lightning impulse breakdown voltage (LI BDV) at 0.2 g/L concentration. Du et al. [24] studied transformer mineral oil-based nanofluids containing TiO_2_ rutile nanoparticles, silicon oil (SO)-treated TiO_2_ and octadecanoic acid (ODA)-treated TiO_2_. The nanoparticles used had an average size of less than 20 nm at concentrations ranging from 0.003 to 0.05 g/mL. They discovered that the optimum concentrations for improving AC BDV depended on nanoparticle surface treatment. The most significant improvement was found in octadecanoic acid-treated TiO_2_ suspensions at a concentration of 0.01 g/mL, which improved by 52.9%. Jin et al. [25] investigated the breakdown voltage features of mineral oil containing dispersed SiO_2_ NPs in two mass fractions and two humidity ranges. The moisture content of the samples had a substantial impact on the BDV values, according to their findings. In the case of samples with higher humidity, an increase in NP concentration raised BDV by 19% and 27% for NP concentrations of 0.01% and 0.02% *w*/*w*, respectively. The BDV augmentation was also detected in materials with a reduced humidity level, but the effect of the NPs was 12 times lower. Saenkhumwong et al. [26] used ZnO nanoparticles of diameter less than 100 nm in two NEOs and CTAB as the surfactant to increase the insulating characteristics. For both types of natural oils, they created five samples with concentrations ranging from 0.01–0.20 g/L. They reported that both types of nanofluids were characterized by increasing BDV values as the concentration of nanoparticles increased. The largest improvements were 47% and 38% for 0.15 g/L (soybean ester) and 0.20 g/L (palm ester), respectively.

Furthermore, another study proved that a small amount (0.001% vol.) of magnetic NPs can lead to significant partial discharge (PD) activity delay [27]. From the aspect of physicochemical properties, Jacob et al. [28] reported a 14.6% thermal conductivity increment after the addition of Al_2_O_3_ NPs into the matrix. Moreover, Fontes et al. [15] noticed a 25% increase in viscosity upon adding diamond NPs, while Ilyas et al. [29] concluded that the dynamic viscosity of alumina NFs decreases with the rise in temperature.

In terms of the mechanisms activated by the suspended NPs under the influence of an external electric field, a variety of models have been proposed [30,31,32,33]. Most of them consider the charged NPs as electron traps caused by the higher permittivity or conductivity of the colloidal suspensions with respect to the base liquid [31]. Consequently, it has been proposed that conductive NPs are more efficient; however, recent studies have demonstrated that semi-conductive or even dielectric NPs can indeed influence the streamer propagation significantly [11,34,35].

Contrary to the number of published research articles on the influence of metal oxide NPs on the aforementioned properties, the effect of metal carbide ones has not been extensively addressed, although it is well known that they are identified by great resistance to oxidation. Consequently, in this study, the influence of commercially obtained SiC NPs on the AC BDV and partial discharge inception voltage (PDIV) of NEO FR3^TM^ is examined. The NPs are integrated in three weight percentage ratios, namely 0.002%, 0.004% and 0.008% *w*/*w*. These concentration levels span a capable range to allow exploring the concentration in the context of optimum AC dielectric strength of the NF. Before the production of uniform colloidal suspensions, the real and imaginary parts of relative permittivity of the NPs have been measured in accordance with the broadband dielectric spectroscopy (BDS) technique. Furthermore, AC BDV and PDIV of the NF samples are measured, processed and compared to the respective ones of the pure base oil. The processing of the results proves that the maximum AC BDV and PDIV enhancement relative to the corresponding ones of the matrix is obtained for the NF filled with 0.004% *w*/*w* SiC NPs by 37.3% and 55.5%, respectively. The theory of electron trapping is utilized analytically in order to explain the BDV improvement, and the results of the BDS technique for the NF samples are adopted to prove that at the highest concentration the dielectric strength is reduced due to an increase in the dielectric constant of the corresponding NF probably because of the existence of quickly generated agglomerates.

## 2. Materials and Methods

This section includes the procedure followed for the dielectric measurements of the under-study NPs and base oil, as well as the synthesis of NFs along with the conduction of experiments of dielectric properties for the NF and matrix samples.

### 2.1. Characterization of the Used Chemicals

**Figure 1:**
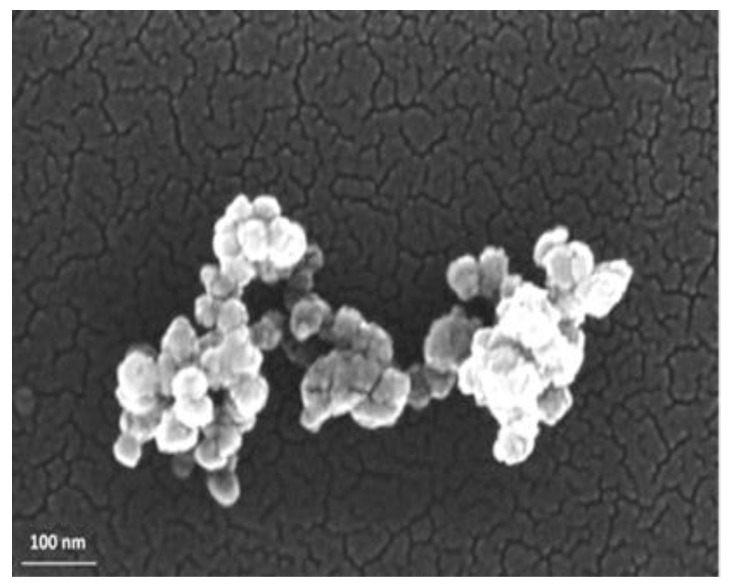
FE-SEM image of SiC NPs at high magnification.

NEO FR3^TM^, obtained by Cargill (Wilmington, DE, USA), is picked as the base liquid. It is readily biodegradable and defined by a density of 0.92 g/mL at 25 °C and good flame resistance as its fire point is greater than 300 °C. The SiC NPs were acquired by NanoAmor in the form of nanopowder with an average nominal diameter in the range of 50 nm and a thermal conductivity of approximately 350 W∙m^−1^/°C at 25 °C. The nominal size of the NPs was ascertained by employing the field emission scanning electron microscopy (FE-SEM) (Zeiss SUPRA 35VP, Oberkochen, Germany) technique, where the NPs were drop-casted by ethanol dispersion onto Si substrates. The FE-SEM depiction of the SiC powder is illustrated in Figure 1, where it is revealed that they indeed have a nominal size.

Concerning the measurements of the relative permittivity ε_r_ and electrical conductivity σ for the above chemicals, the BDS technique is exploited [36]. Each sample is put in a cylindrical capacitor. Then, the liquid sample cell is mounted into the standard Novocontrol sample cell (16 mm diameter) and an AC voltage of 1 V rms is applied for the calculation of relative permittivity *ε_r_* in complex form as indicated by Equation (1), from a Novocontrol Alpha analyzer (Novocontrol Technologies GmbH & Co., Frankfurt, Germany) for a frequency range of 0.1 Hz to 1 MHz:

(1) $$\varepsilon_{r}=\varepsilon_{r}{}^{\prime }-j\varepsilon_{r}{}^{\prime \prime }\;$$

where *ε_r_*^′^ is the real part of relative permittivity expressing the dielectric constant and *ε_r_*^″^ is the imaginary part, which expresses the dielectric losses contributed by the leakage current and dielectric polarization.

On account of the electrical conductivity, it is calculated from the imaginary part of relative permittivity from Equation (2):

(2) $$\sigma =\omega \cdot \varepsilon_{0}\cdot \varepsilon_{r}{}^{\prime \prime }\;$$

where *ω* is the angular frequency in rad/s and *ε*_0_ is the permittivity of vacuum in F/m.

**Figure 2:**
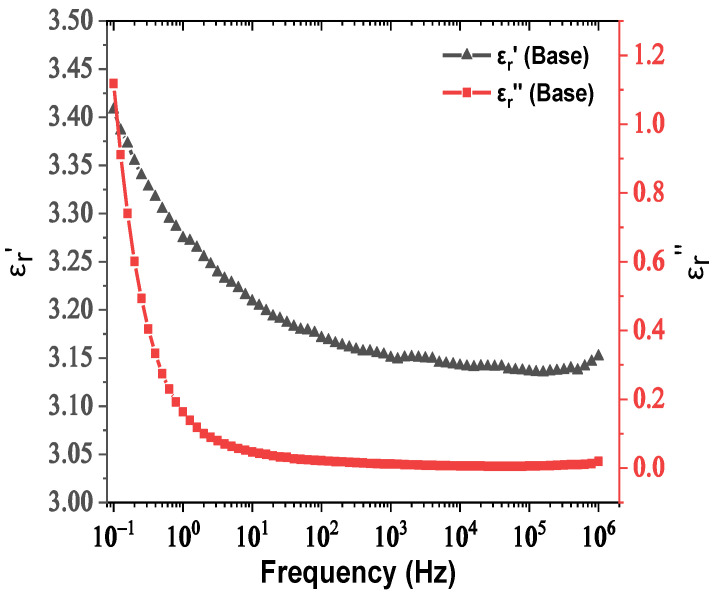
Change in the real and imaginary parts of relative permittivity versus frequency for natural ester oil.

**Figure 3:**
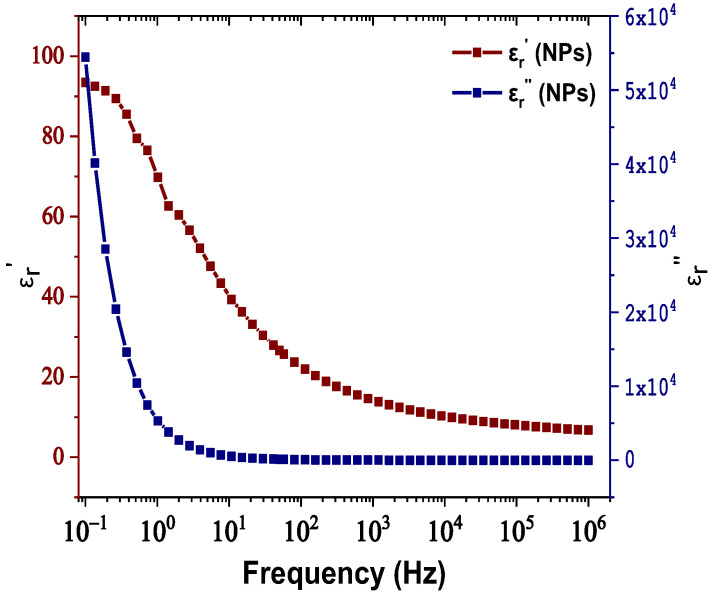
Change in the real and imaginary parts of relative permittivity versus frequency for the SiC nanoparticles.

**Figure 4:**
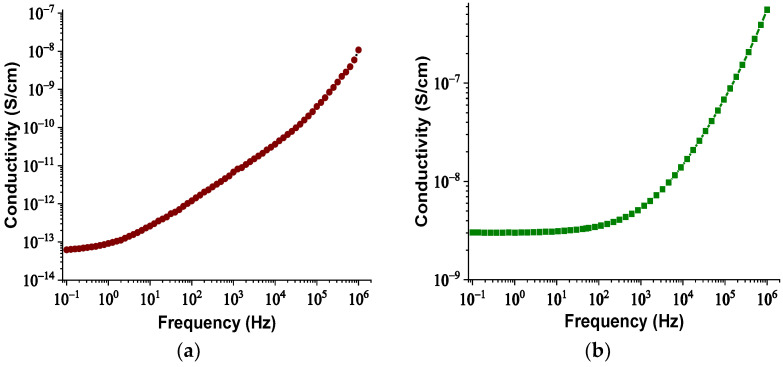
Change in electrical conductivity versus frequency: (**a**) for the matrix sample and (**b**) for the SiC nanoparticles.

The real and imaginary parts of relative permittivity as a function of frequency for the matrix and NPs are presented in Figure 2 and Figure 3, respectively. Figure 4 depicts the electrical conductivity of base oil and the SiC NPs accordingly. For the measurement of the dielectric dissipation factor at 90 °C, the samples are evidenced isothermally, with the temperature controlled by a Quatro system within ±0.2 °C.

### 2.2. Preparation and Synthesis of Nanofluid Samples

A 450 mL volume of NEO is decided to be the base for the synthesis of the three NF samples. In order to eliminate the negative effects of impurities and moisture on BDV, each sample is filtered and dried in a hot air oven at 120 °C for 24 h. The Karl–Fischer titration method is used to confirm that the remaining moisture is within the recommended limits [37,38]. The moisture level is measured with Mitsubishi Chemicals Co. (Tokyo, Japan) CA-100 moisture meter (Tokyo, Japan), and it is confirmed by Metrohm KF Coulometer 831 that it is below 200 ppm according to the ASTM D1533-00 standard [39]. Afterwards, appropriate amounts of SiC powder are immersed in the base to obtain the desired 0.002%, 0.004% and 0.008% *w*/*w* concentrations, respectively. The identification of the samples in question is exhibited in Table 1.

**Figure 5:**
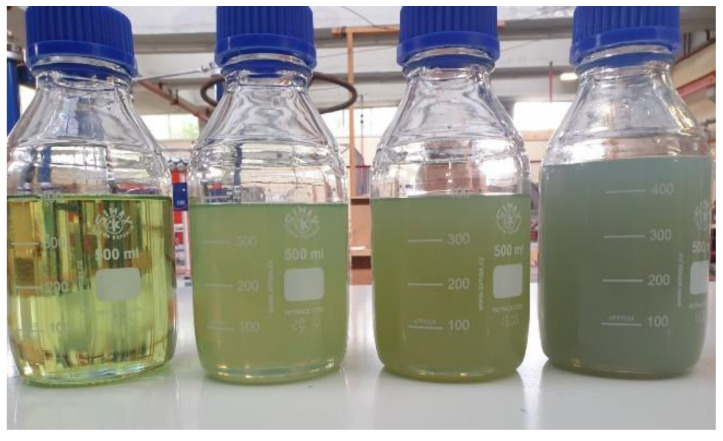
The samples immediately after their production, from left to right: Net, NF1, NF2 and NF3 samples.

Due to the high surface areas of NPs per unit volume, the colloidal suspensions inside the host liquid tend to agglomerate shortly after their addition, under the influence of van der Waals interaction forces forming clusters [11,12,18,37,38,40]. Hence, in order to ensure the distribution of uniform colloidal suspensions, the three NF samples are subjected to ultrasonication for 1.5 h in total at the ultrasonic cleaner Elmasonic S 40 H (Frankfurt, Germany) purchased by Elma (Frankfurt, Germany). Specifically, the water inside the ultrasonic bath is replaced every 30 min to prevent the samples from overheating. This cycle is repeated two times for each sample to establish a uniform dispersion of the colloidal suspensions, as indicated in Figure 5, where the samples are depicted shortly after their homogeneous synthesis. In the same figure, a base oil sample is also presented as long as it is stressed under the same experiments for the sake of comparison. The color difference between the matrix and the NF samples is obvious, as well as the color difference among the NF samples as we proceed from the NF with the lowest to the one with the highest NP weight percentage ratio.

### 2.3. Experimental Procedure

The freshly produced NF samples, along with a base oil sample, are stressed under AC voltage in order for their AC BDV to be determined. The AC BDV experiment is the most important destructive dielectric test, as it measures the ability of the dielectric liquid to withstand AC electric stress, considering that the power transformer regularly operates under AC voltage. It is well known that the particle concentration inside the base may differ significantly over time owing to the colloids’ aggregation and finally sedimentation effects [11,41]. For this reason, the experiments take place shortly (within the first 48 h) after the preparation of the samples in order to eliminate the influence of NPs’ agglomeration over time on our results.

**Figure 6:**
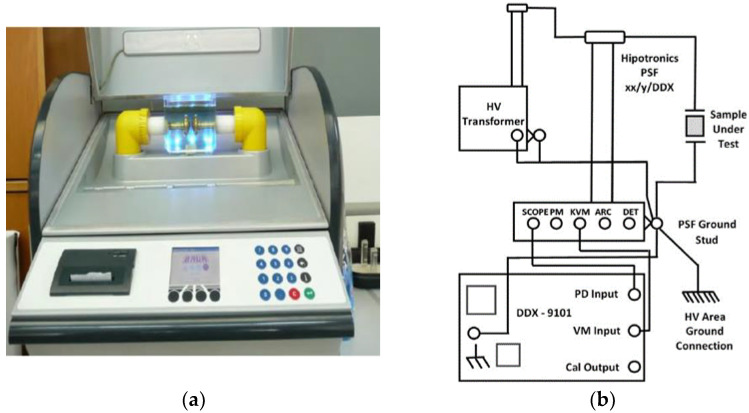
AC BDV and PD experimental devices: (**a**) The BAUR DTA generator with the testing cell; (**b**) the schematic diagram of the PD setup.

For the conduction of the AC BDV measurements, each one of the four samples is stressed under increasing AC voltage using a Baur DTA 100 C generator (Figure 6a) in consonance with the IEC 60156 standard. A transformer increases the voltage level up to 100 kV with a rate of 2 kV/s, until breakdown occurs between two Rogowski electrodes of 13 mm diameter, with their gap fixed at 2.5 mm. In total, 36 breakdown events are collected for each sample and their mean AC BDV, and consequently their dielectric strength, is estimated. As long as the BDV is a random variable, the results are also fit to the Weibull distribution to assess their BDV in significant probability levels for the reliability of the insulation and, by extension, the power transformer [11,22,35,42].

Furthermore, the efficiency of the combination of oil-paper insulation is evaluated with the non-destructive PD test. Beyond that, defining the PD activity in dielectric fluids is very important to retain suitable kinds of NPs inside the liquid insulation to suppress the PD activity and therefore delay the aging process of the oil [43,44]. The paper impregnation lasted about 5 min and took place at a temperature range of 20–25 °C, a humidity of about 10 g/m^3^ and atmospheric pressure approximately equal to 758 mm Hg. Regarding the conduction of the experiment, a High Voltage test transformer (HIGH VOLT GmbH Transformer PEOI 40/100 100kV) is used, with a TETTEX Instruments PD Detector DDX-9101 PD detector measuring the apparent charge to a HIPOTRONICS capacitor PSF 100-1 nominal capacitor (Figure 6b). The test cell consists of two plate electrodes with the gap between them set at 0.75 mm. Nomex^®^ Dupont^TM^ insulating paper of 0.75 mm thickness impregnated with the base and NF samples is placed in the gap and stressed under AC voltage with an increasing rate of 0.1 kV/s. As for PDIV, it is regarded as the level of voltage that is characterized by an apparent charge accumulation greater than the threshold value of 10 pC [11,40,41]. Each experiment is repeated four times to increase the reliability of the obtained results and the mean value is considered for the performance analysis.

## 3. Results

### 3.1. AC Breakdown Voltage

**Figure 7:**
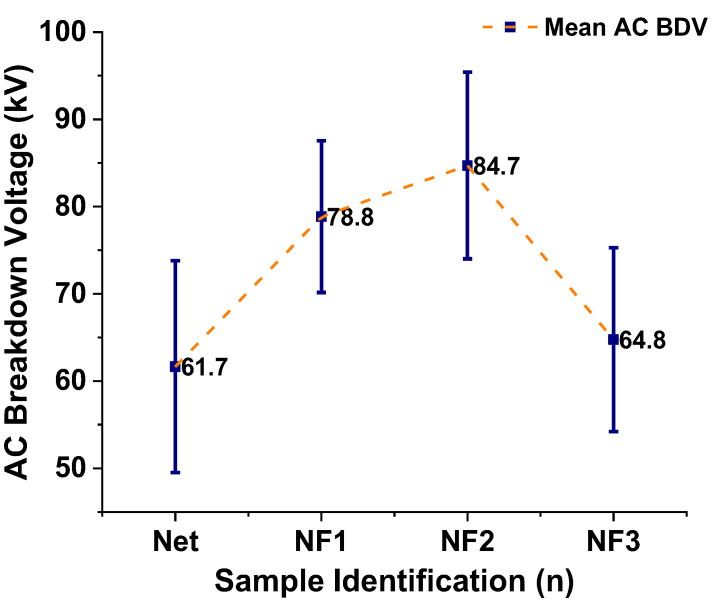
Mean values of the AC BDV along with error bars for the samples in question.

Based on the collected 36 BD events for each sample stressed under AC voltage, the mean value of them (mean AC BDV) and the standard deviation of the results are calculated and presented in Table 2 and graphically in Figure 7. It is obvious that the dielectric strength increases with an increase in the NP loading until the 0.004% *w*/*w* level (NF2 sample), where the highest improvement with respect to the NEO is noticed, by 37.3%. From this point, doubling the NP concentration (NF3 sample) results in a rapid decrease in the BD performance, which becomes similar to the base oil sample’s performance.

In order for the failure probabilities in specific important levels to be estimated, the Anderson–Darling goodness-of-fit test is performed to check if the data follow the Weibull distribution. The Weibull distribution is a popular statistical law to adjust the experimental AC BDV results because it does not make assumptions on skewness and kurtosis [22,35]. The cumulative distribution function (CDF) of the Weibull distribution is expressed by Equation (3):

(3) $$F_{\left(x\right)}=1-e^{-{\left(\frac{x}{\lambda }\right)}^{k}}\;$$

where *x* is the AC BDV, *λ* is the scale parameter and *k* is the shape parameter. The *p*-value, utilizing the Anderson–Darling test, is calculated as follows: Firstly, the Anderson–Darling (AD) statistic is calculated from Equation (4):

(4) $$AD=-n-\frac{1}{n}\overset{n}{\underset{i=1}{\sum }}\left(2i-1\right)[lnF\left(x_{i}\right)+\ln\left(1-F\left(X_{n-i+1}\right)\right)]$$

where *n* is the number of measurements, *F*(*x*) is the CDF of the Weibull distribution and *i* is the *ith* measurement, calculated when the data are sorted in ascending order. After calculating the *AD* statistic, the *p*-value can be estimated from (5), depending on the *AD* value.

(5) $$p-\mathrm{value}=\{\begin{array}{c} \exp\left(1.2937-5.709\left(AD\right)+0.0186\right){\left(AD\right)}^{2}),\;AD\geq 0.60\\ \exp\left(0.9177-4.279\left(AD\right)-1.38{\left(AD\right)}^{2}\right),\;0.34<AD<0.60\\ 1-\exp\left(-8.318+42.796\left(AD\right)-59.938{\left(AD\right)}^{2}\right),\;0.20<AD<0.34\\ 1-\exp\left(-13.436+101.14\left(AD\right)-223.73{\left(AD\right)}^{2}\right),\;AD\leq 0.20 \end{array}$$

**Figure 8:**
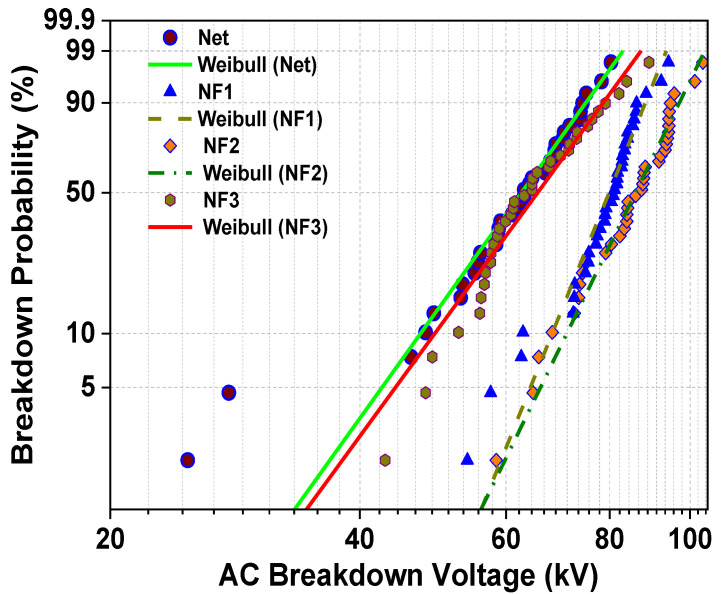
Probability density plot of the BD events with conformity to the Weibull line.

According to statistics, if the *p*-value is higher than the significance level, there is not enough evidence to reject the hypothesis that the data follow a distribution. For this reason, a significance level α is picked (α = 0.05), and from the Anderson–Darling test, the *p*-value for the Weibull distribution is calculated in terms of the obtained results of all the samples and compared to the significance level (Table 3). As is apparent from Table 3, the *p*-value is higher than the significance level for all occasions, and consequently the whole data obey the Weibull distribution. In Figure 8, the probability density plot is depicted, where it is shown how the breakdown events are fit to the corresponding Weibull line. To produce this plot, the scale and shape parameters are necessary and are also presented in Table 1. The slope of the linear fit characteristic is revealed by the shape value, whereas the scale value indicates the BDV with a failure probability of 63.2% and in units of kV.

From this plot, the BDV at failure probabilities of 1%, 10% and 50% are calculated and presented in Table 4. The choice of these levels corresponds to their importance for the operation of the HV equipment. U_50%_ is an indication of the expected BDV, U_10%_ provides an indication of the lowest possible BDV, and therefore provides information on the reliability of the insulation [11,12,22,34,35,37,38], while U_1%_ corresponds to the limit of voltage for safety and continuous operation of the transformer [11,22], a critical parameter for the insulation coordination of power systems under lightning and surge events [45,46].

From the processing described above, it is clear that the addition of SiC NPs in weight percentage ratio until a concentration level of 0.004% can lead not only to the expected AC BDV augmentation but also to an improvement of U_10%_ and U_1%_ with a maximum increase of 50.1% and 65.2%, respectively, a fact that could ensure more reliable operation of the power transformer due to the delay of the streamer initiation and transition to faster modes [22,34,35,47].

### 3.2. Partial Discharge Test

**Figure 9:**
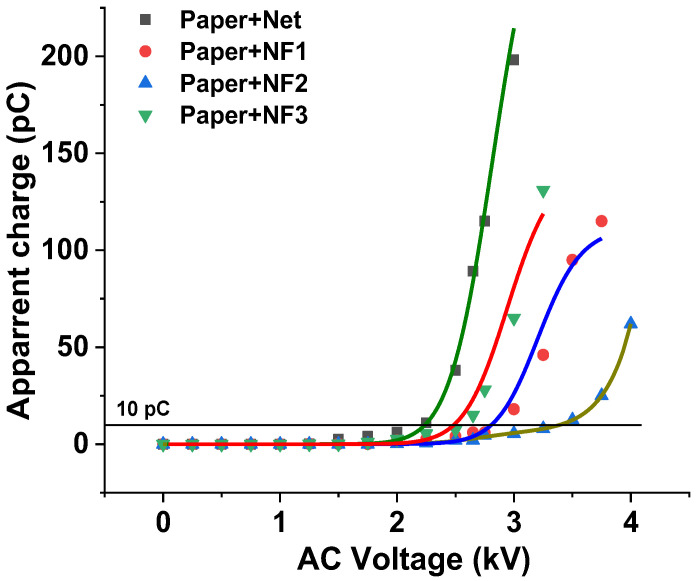
Apparent charge versus the applied AC voltage for the insulating paper impregnated with the Net and NF samples.

The change in the accumulated apparent charge versus the applied AC voltage for the insulating paper impregnated with the under-study samples is demonstrated in Figure 9 for an AC voltage rate, showing that the apparent charge accumulation does not exceed the value of 200 pC. Each symbol corresponds to the value of the apparent charge at a specific voltage level, which is the mean value of the four -times repeated experiment at the same voltage level. The threshold value of 10 pC, where the PDIV is considered, is also indicated in the same figure. By means of this depiction, the PDIV of the paper impregnated with NF2 shows an enhancement of 55.5% with respect to the paper impregnated with the Net sample. Furthermore, as observed with the AC BDV experiment, the resistance to the appearance of PD of the combination of Nomex^®^ Dupont^TM^ insulating paper and the NF3 sample is similar to that of the paper impregnated with the matrix.

**Figure 10:**
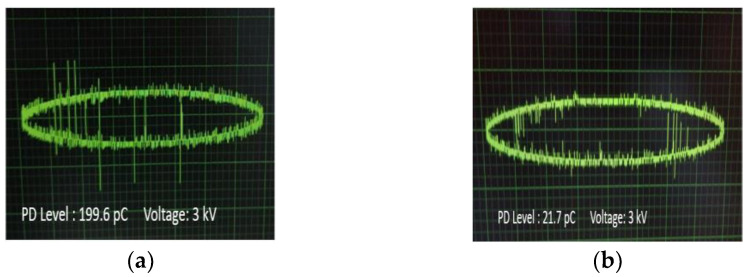

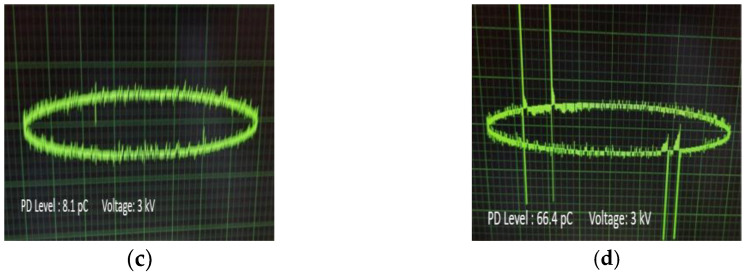
Waveforms of PD pulses at 3 kV AC voltage for the insulating paper impregnated with: (**a**) Net; (**b**) NF1; (**c**) NF2; (**d**) NF3 samples.

In Figure 10a–d, the PD levels in the form of pulses are demonstrated for each occasion at the same level of AC voltage (3 kV) versus the phase of the applied AC voltage, where it is obvious that the peak charge in the impregnated papers to Net and NF3 is higher than the corresponding ones of impregnated papers to NF1 and NF2. The multiple pulses of irregular form, as are obvious for the Net and NF3 impregnated insulating paper, indicate the presence of voids in their vicinity, which could be an indication of a faster aging process.

### 3.3. Comparison of Dielectric Dissipation Factor

**Figure 11:**
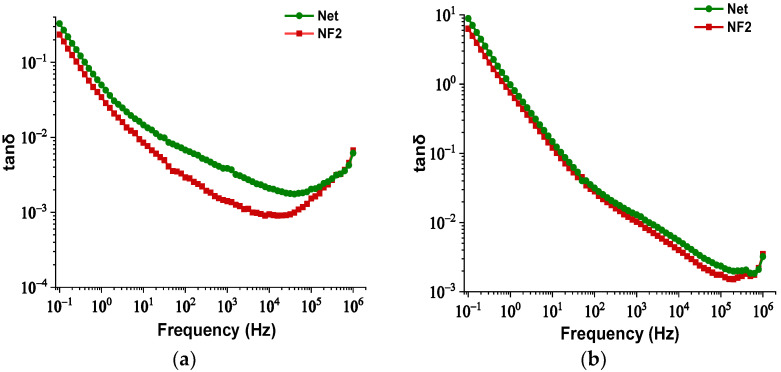
Change in dielectric dissipation factor versus frequency of the Net and NF2 samples: (**a**) at 25°C and (**b**) at 90 °C.

As discussed in the Section 1, one of the disadvantages of the VO is the increased dielectric dissipation factor (tanδ). Therefore, it is of major importance to monitor this electrical parameter for the NF containing the optimal NP concentration, in terms of AC BDV and PDIV maximization. In Figure 11a,b, the change of tanδ is illustrated as a function of frequency for both samples at 25 °C and 90 °C, respectively. It is evident that the integration of this kind of NPs leads to a reduction of this factor under both temperature conditions, which is an indication of lower dielectric losses in comparison to the bulk.

## 4. Discussion

Based on the mean AC BDV data, it has been shown that adding semi-conducting SiC NPs to the matrix improves the mean AC BDV, with NF2 having the highest maximization, and that doubling the NP’s loading decreases the AC BDV. Many other published studies in the field [11,12,13,22,34,47] have discovered a similar pattern. The influence of these semi-conductive carbide NPs on the increase of AC BDV up to an optimal concentration can be explained by the fact that the NPs up to this weight percentage ratio could boost the shallow trap density in the NF, causing streamer propagation to be delayed due to electron trapping and de-trapping in shallow traps [30,31,32,33].

The shallow trapping model will be employed to clarify the mechanisms activated by the NPs to trap the fast electrons at the tip of the streamer and slow down its propagation between the electrodes. According to [30,31], under the influence of an external applied electric field, if there is reasonable discrepancy in the real part of relative permittivity between the integrated NP and base oil, then induced or polarized charges are generated at their interface. These charges result in the production of a potential well that can trap the fast electrons at the tip of the streamer in shallow traps and slow down its propagation; consequently, a higher electric field value should be applied in order for the electrons to be released and bridge the gap. With reference to the SiC NPs, because of their characterization as semiconductors, the potential well is developed due to polarization [31] and is expressed by Equation (6) for the electric field direction:

(6) $$\varphi_{SiC}{}_{\left(r\right)}=\frac{{\varepsilon_{2}}^{\prime }-{\varepsilon_{1}}^{\prime }}{2{\varepsilon_{1}}^{\prime }+{\varepsilon_{2}}^{\prime }}R^{3}E_{0}\frac{1}{r^{2}},\;r\geq R\;$$

where *ε*_1_′, *ε*_2_′ are the real parts of the permittivities of the base and NPs in F∙m^−1^, respectively, *R* is the radius of the suspended NPs and *E*_0_ is the value of the external electric field in V∙m^−1^.

Moreover, the deposited charge in each suspended NP due to the formation of the above potential well is expressed as Equation (7):

(7) $$Q_{\left(t\right)}=Q_{s}{}_{SiC}\frac{\frac{t}{\tau_{pc}}}{1+\frac{t}{\tau_{pc}}}\;$$

where *t* is the time of charging, $Q_{s}{}_{SiC}$ is the total charge that can be captured by each charged NP, i.e., the saturation charge, and is calculated for this case from Equation (8) [31]:

(8) $$Q_{s}{}_{SiC}=-12\pi {\varepsilon_{1}}^{\prime }{Ε}_{0}R^{2}\frac{{\varepsilon_{2}}^{\prime }}{2{\varepsilon_{1}}^{\prime }+{\varepsilon_{2}}^{\prime }}\;$$

and *τ_pc_* is the time constant for NP charging, which is expressed by Equation (9):

(9) $$\tau_{pc}=\frac{4{\varepsilon_{1}}^{\prime }}{\rho_{e}\mu_{e}}\;$$

where *ρ_e_* is the electron uniform charge density and *μ_e_* is the electron mobility.

**Figure 12:**
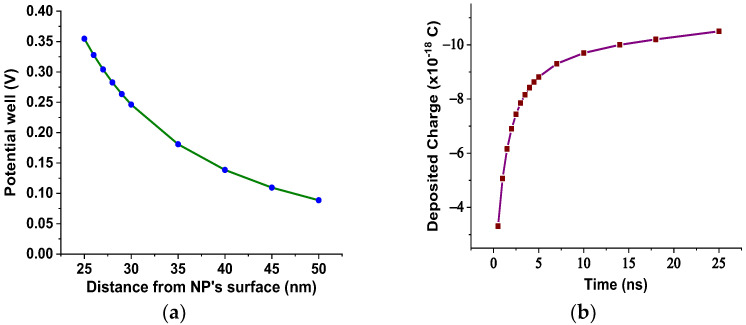
(**a**) Potential well distribution versus the distance from the SiC NP surface for E_0_= 20 kV∙mm^−1^; (**b**) deposited charge in each SiC NP versus time for E_0_= 20 kV∙mm^−1^.

In our case, based on the results of Figure 3, the SiC NPs have dielectric constant equal to 26.6 at a rated frequency of 50 Hz, contrary to the matrix, which is characterized by a dielectric constant of 3.2 at 50 Hz according to Figure 2. The important divergence in their relative permittivities leads to the formation of a potential well as a function of the distance from their surface, which is described by Equation (3) and depicted in Figure 12a for electric field strength *E*_0_ = 20 kV∙mm^−1^. From this depiction, it is obvious that the high potential well distribution, especially as we approach the NPs’ surface, is able to catch the charge carriers, especially electrons, due to their higher mobility. The electron trapping takes place at a fast rate, as shown in Figure 12b, where it appears that half of the saturation charge per NP is deposited in about 1.1 ns in shallow traps, obtained by using Equation (4), a fact that explains the increased dielectric performance with the integration of these kinds of NPs. This mechanism leads to an enhancement of AC BDV, because of the much slower mobility of NPs with respect to electrons. In terms of (7), the saturation charge of each suspended SiC NP is −1.08×10^−17^ C (67 e^−^). Therefore, each SiC NP under the influence of an external electric field of 20 kV∙mm^−1^ can trap about 67 electrons at a very fast rate and thus modify the fundamental electrohydrodynamics of the streamer propagation, leading to improved AC BDV.

In an effort to explain the negative effect on AC BDV regarding NF3, the Derjaguin–Landau–Verwey–Overbeek (DLVO) theory is considered [34]. Under the influence of an increasing external electric field, like the one in the AC BDV test, the oil molecules can lose electrons due to impact ionization through collisions with the highly energetic electrons injected from the electrodes. In this way, these oil molecules become positively charged and are called positive ions. Subsequently, some of the free electrons in the vicinity of the liquid are attached to neutral oil molecules, charging them negatively. These negatively charged molecules are called negative ions. When the colloids are charged in the base oil, they attract the positive ions, namely counter-ions, which form a rigorous layer around each colloid; this layer of counter-ions is known as the Stern layer [34]. Besides, the negative ions (namely co-ions) along with additional positive ions are repelled by the negative colloid and the Stern layer respectively. Both kinds of ions form a second layer around the Stern layer, until reaching equilibrium, which is called the diffuse layer [34]. Both layers compose the model of the formation of the electrical double layer (EDL) around each charged NP.

According to the DLVO theory, the total interaction between two NPs added in a liquid medium is the combination of the van der Waals attraction force and the electrostatic repulsion force caused by their EDLs. The van der Waals attraction force, considering the NPs as spherical with the same radius R at the time of their integration into the matrix, is expressed by Equation (10):

(10) $$W_{\left(D\right)}=-\frac{AR}{12D}\;$$

where *A* is the Hamaker constant and *D* is the separation distance between them.

**Figure 13:**
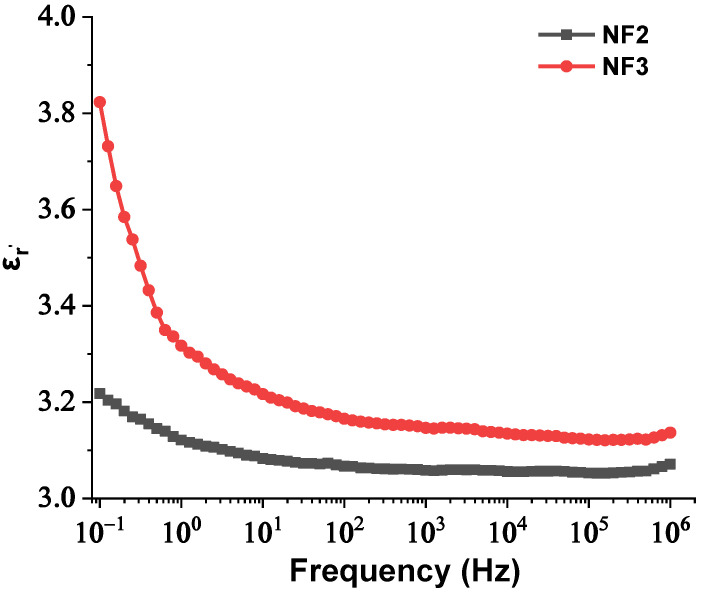
Change in the real part of relative permittivity versus frequency of the NF2 and NF3 samples.

**Figure 14:**
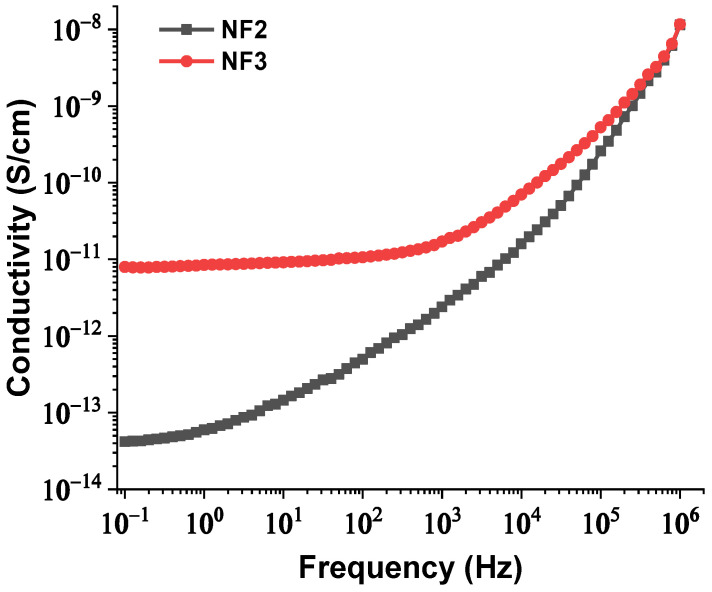
Change in electrical conductivity versus frequency of the NF2 and NF3 samples.

Hence, with an increase in the NPs’ concentration from 0.004% to 0.008% *w*/*w*, the separation distance of them declined, which resulted in overlap of some of the NPs’ EDLs due to extremely augmented van der Waals forces. The rapid aggregation could have led to the formulation of conductive bridges between the electrodes and hence a reduction in the AC BDV. In order for this theory to be verified, the BDS technique was used to measure the dielectric constant and electrical conductivity of the NF2 and NF3 samples just after their preparation. The change in their dielectric constants and electrical conductivities versus frequency is depicted in Figure 13 and Figure 14, respectively. It is evident that at 50 Hz frequency, NF2 has a lower dielectric constant and a much lower electrical conductivity than NF3. The increase in electrical conductivity of NF3 can be attributed to the mechanism described above in detail. So, the results of the BDS technique confirm the AC BDV results and consequently the developed theory.

The results of the performed PD test also verify the hypothesis that the NPs’ agglomeration is the main cause for the existence of an optimal concentration in terms of AC BDV performance. It was found that the resistance to the appearance of PD is maximized when the paper impregnated with the NF2 sample is stressed and then it falls when the combination of solid insulation with NF3 is tested. According to [43], two factors affect the resistance to PD: firstly, the ability of the SiC NPs to act as electron traps, based on the shallow trapping theory as explained before. The EDL plays a pivotal role as well; however, in our case the PD experiment is performed in the insulating paper impregnated with the samples; therefore, the influences of the EDL and its diffuse layer are neglected.

## 5. Conclusions

In this paper, the influence of SiC NPs on the AC BDV and PDIV of NEO FR3 was investigated. The NPs were added in three weight percentage ratios, namely 0.002%, 0.004% and 0.008% *w*/*w*, synthesizing the NF1, NF2 and NF3 samples respectively. The processing of the results indicated that the highest AC BDV and PDIV improvement in comparison to the base oil’s characteristics was obtained for the NF2 sample, by 37.3% and 55.5%, respectively. The statistical analysis confirmed that the same sample also demonstrated increased BDV at the 10% probability level after fitting to the Weibull distribution, by 50.1% with respect to the matrix. This increase was attributed to the ability of the SiC NPs to trap the charge carriers through the production of a potential well on their interface caused by polarized surface charges. The BDS technique was adopted to show that the dielectric constant and electrical conductivity of NF3 had been augmented to prove that the overlapping of the NPs’ EDLs due to reduction of their separation distance creating conductive paths was responsible for the existence of an optimal concentration.

In addition, the improved behavior of the insulating paper impregnated with NF2 has been attributed to the ability of the SiC NPs to operate as electron traps and finally increase the resistance to the appearance of PD. Last but not least, the NF containing this amount of NPs demonstrated a decreased dielectric dissipation factor for a frequency range of 0.1 Hz–1 MHz at both 25 and at 90 °C.

## Figures and Tables

**Table 1:** Identification of the samples.

Sample (n)	NP Concentration (% *w*/*w*)
Net (Base)	0
NF1	NF with 0.002% SiC NPs
NF2	NF with 0.004% SiC NPs
NF3	NF with 0.008% SiC NPs

**Table 2:** Descriptive statistics of the liquid samples under investigation.

Sample (n)	Mean AC BDV (kV)	Standard Deviation (kV)	Scale (kV)	Shape
Net	61.7	12.1	66.1	6.7
NF1	78.8	8.7	82.4	11.9
NF2	84.7	10.7	89.2	9.9
NF3	64.8	10.5	69.2	6.6

**Table 3:** Anderson–Darling goodness-of-fit test for the Weibull distribution.

Sample (n)	*p*-Value
Net	0.25
NF1	0.13
NF2	0.25
NF3	0.18

**Table 4:** Withstand voltages at 1, 10 and 50% failure probability levels with fitting to the Weibull distribution.

Sample (n)	U_50%_ (kV)	U_10%_ (kV)	U_1%_ (kV)
Net	62.6	47.3	33.9
NF1	80.5	69.9	58.7
NF2	85.9	71.0	56.0
NF3	65.5	49.3	34.5

## Data Availability

The data presented in this study are available from the corresponding author upon request. The data are not publicly available due to privacy.
